# Is 3D faster and safer than 4K laparoscopic cholecystectomy? A randomised-controlled trial

**DOI:** 10.1007/s00464-019-06958-w

**Published:** 2019-07-18

**Authors:** Matt Dunstan, Ralph Smith, Katie Schwab, Andrea Scala, Piers Gatenby, Martin Whyte, Tim Rockall, Iain Jourdan

**Affiliations:** 1 Royal Surrey County Hospital, Minimal Access Therapy Training Unit (MATTU), Leggett Building, Daphne Jackson Road, Manor Park, Guildford, Surrey GU2 7WG UK; 2 Faculty of Health and Medical Sciences, University of Surrey, Guildford, Surrey GU2 7XH UK

**Keywords:** Laparoscopy, Cholecystectomy, Depth Perception, Imaging, Three-Dimensional

## Abstract

**Background:**

Laparoscopic surgery has well-established benefits for patients; however, laparoscopic procedures have a long and difficult learning curve, in large part due to the lack of stereoscopic depth perception. Developments in high-definition and stereoscopic imaging have attempted to overcome this. Three-dimensional high-definition (3D HD) systems are thought to improve operating times compared to two-dimensional high-definition systems. However their performance against new, ultra-high-definition (‘4K’) systems is not known.

**Methods:**

Patients undergoing laparoscopic cholecystectomy were randomised to 3D HD or 4K laparoscopy. Operative videos were recorded, and the time from gallbladder exposure to separation from the liver (minus on table cholangiogram) was calculated. Blinded video assessment was performed to calculate intraoperative error scores.

**Results:**

One hundred and twenty patients were randomised, of which 109 were analysed (3D HD *n* = 54; 4K *n* = 55). No reduction in operative time was detected with 3D HD compared to 4K laparoscopy (median [IQR]; 23.41 min [17.00–37.98] vs 20.90 min [17.67–33.03]; *p* = 0.91); nor was there any decrease observed in error scores (60 [56–62] vs 58 [56–60]; *p* = 0.27), complications or reattendance. Stone spillage occurred more frequently with 3D HD, but there were no other differences in individual error rates. Gallbladder grade and operating surgeon had significant effects on time to complete the operation. Gallbladder grade also had a significant effect on the error score.

**Conclusions:**

A 3D HD laparoscopic system did not reduce operative time or error scores during laparoscopic cholecystectomy compared with a new 4K imaging system.

**Electronic supplementary material:**

The online version of this article (10.1007/s00464-019-06958-w) contains supplementary material, which is available to authorized users.

The benefits of laparoscopic over open surgery include quicker recovery, and reduced pain, blood loss and wound infection [1–3]. However, laparoscopic surgery is technically challenging, due to the reduced tactile sensation and degrees of freedom of the instruments, the altered ergonomics, lack of camera stability and loss of binocular depth perception [4, 5]. In particular, overcoming the loss of stereoscopic depth perception is associated with a long learning curve [6, 7]. Inaccurate object localisation and depth perception in laparoscopic surgery may be dangerous [8]. Advances in video technology, namely high-definition two-dimensional imaging (2D HD) and stereoscopic (three-dimensional) laparoscopes (3D HD), have been developed in an attempt to reduce complication rates and to shorten the learning curve. It has been suggested that the introduction of 2D HD and 3D systems have improved depth perception [9], by enhancing monocular and binocular depth-perception cues, respectively [10]. A systematic review of simulator-based studies has suggested that surgeons complete tasks more quickly and with fewer errors when using dual-channel, passive polarising stereoscopic systems, compared to that when using 2D HD systems [11]. Recently, the European Association for Endoscopic Surgery (EAES) has published recommendations that 3D systems should be utilised in the clinical setting to decrease operating times [12]. However, these systems are expensive, and around ten percent of surgeons cannot perceive stereoscopic depth [13, 14]. Furthermore, no clinical trials have investigated whether 3D systems have performance benefits over ultra-high-definition (‘4K’) laparoscopic imaging—a new two-dimensional technology with four times the number of pixels of HD, which potentially provides stronger monocular depth-perception cues.

We hypothesised that the use of a 3D HD laparoscopic system would decrease the duration of surgery and the error score during laparoscopic cholecystectomy, when compared to a 4K system.

## Materials and methods

A randomised-controlled trial was conducted, with the primary outcome being time to complete laparoscopic cholecystectomy. A power calculation was based on a median operating time for laparoscopic cholecystectomy of 47 min (range 36–64 min) [15, 16]. Based on this operating time, a maximum of five laparoscopic cholecystectomies can be scheduled into a full-day operating list. A reduction in operating time of 12 min in each of five operations would allow an extra hour of operating, and the addition of a further operation to the list. A 12-min difference was therefore deemed clinically significant. With 46 patients in each treatment arm, the study had 80% power to detect a 12-min (25%) difference in operative time, with statistical significance declared if *p* < 0.05. Target recruitment was set to 60 patients in each arm, to allow for exclusions and equipment failure. The secondary outcomes were the Technical Skills Checklist error score [17], and 30-day complication and reattendance rates. Inclusion criteria were: patients aged 18 to 85 undergoing laparoscopic cholecystectomy with or without on table cholangiogram. Exclusion criteria were: conversion to open surgery or previous major upper abdominal surgery. All patients gave informed, written consent. The three operating consultant surgeons had previous experience of 3D laparoscopic cholecystectomy, and had been screened for stereoacuity, visual acuity and colour vision. A standard ergonomic arrangement was used. Four-port laparoscopic cholecystectomy was performed using either a 3D HD or a 4K laparoscopic system. The 3D stack was the dual-channel passive polarising Image1 S 3D high-definition system (Karl Storz, Tuttlingen, Germany) with a 45.9 inch JVC passive polarising LCD screen (JVC, Yokohama, Japan; although for the first four 3D cases the standard 31 inch stack display was used, which was felt unlikely to introduce significant bias). The 4K system was the VISERA 4K UHD system (Olympus Europa, Hamburg, Germany) with a 55 inch Sony LCD screen (Sony, Tokyo, Japan).

**Fig. 1:**
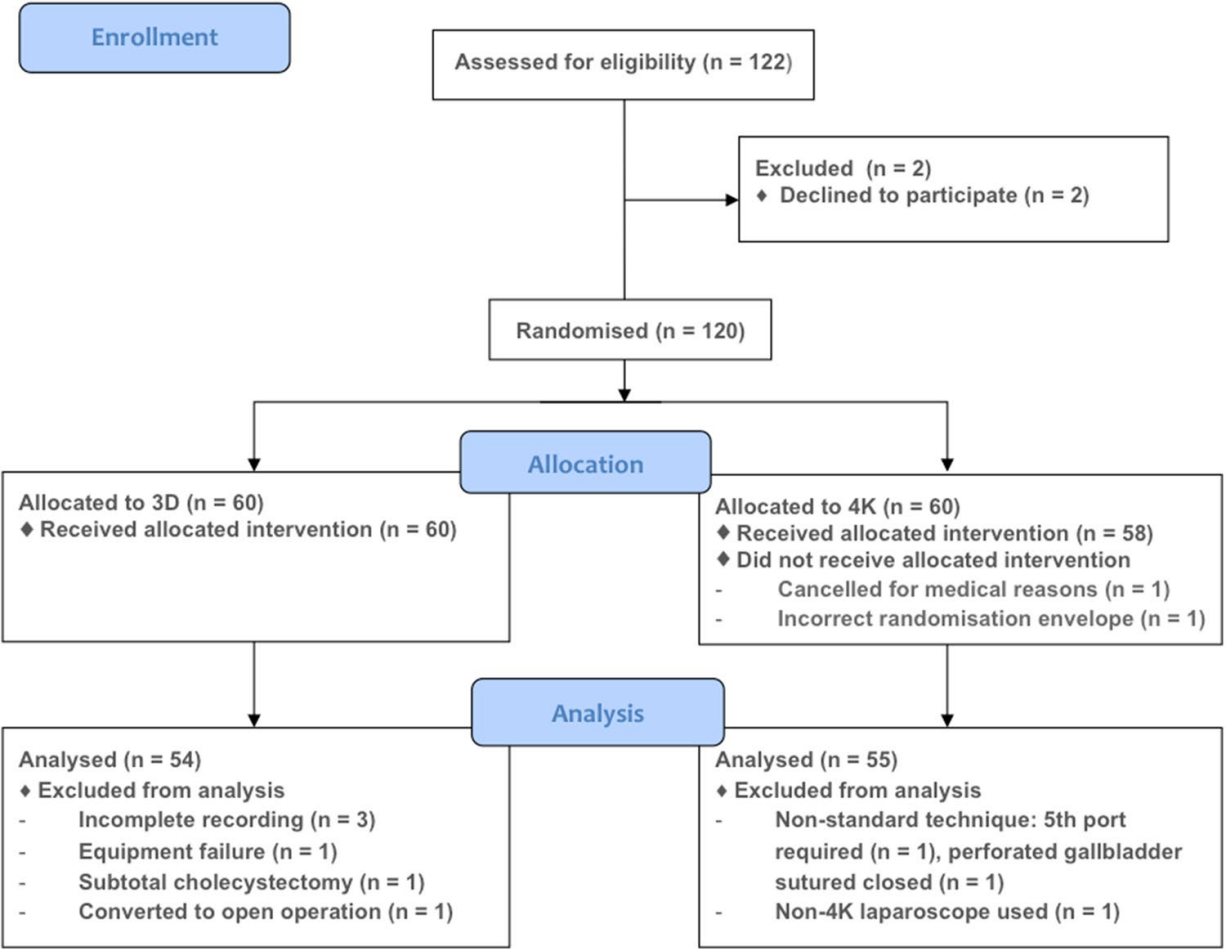
Consort diagram

Randomisation was performed with variable block sizes, and the allocation process was double-blinded using a sealed envelope method. Participants were recruited between September 2016 and September 2017. The principal investigator recruited and allocated participants, and was then unblinded.

Operative videos were anonymised, converted to 2D HD, and edited to remove overlays/watermarks by the Principal Investigator using Final Cut Pro X (Apple Inc., Cupertino, California, USA), in order to blind assessors to the system used. The videos were edited to begin at the first attempt to expose the gallbladder (including adhesiolysis) to complete separation from the liver. If on table cholangiogram was performed, this was cut from the edit. The time to complete cholecystectomy was then derived from the length of the video. Operations were graded for difficulty [18] as: Grade 1 (no adhesions or fat over Calot’s triangle, and normal anatomy), Grade 2 (loose adhesions, obstructed view of cystic anatomy e.g. by fat, or BMI > 30) or Grade 3 (dense adhesions, duodenum or bile duct adherent to gallbladder, active inflammation, contracted gallbladder, empyema, dense adherence to liver, difficult abnormal or unclear anatomy, or gallstone in Hartmann’s pouch or gallbladder neck). Videos were graded by the Principal Investigator in consensus with a blinded surgeon who had not been involved in the trial. This surgeon also conducted the error assessment using the Technical Skills Checklist error score [17]. This involved calculating a weighted score for minor (bile spillage, diathermy burn to liver, incomplete clipping, fallen clip, delay in identifying Calot’s triangle anatomy), major (stone spillage, injury causing liver bleeding, injury to cystic artery or duct, loss of pneumoperitoneum), and significant major (major vessel or major duct injury, other visceral injury) errors. Progressively higher scores were given depending on whether each error was not committed, committed and corrected, or committed and not corrected. In addition, the more serious the error, the greater the weighting (see Supplement 1). A total error score was calculated from the sum score for each error type. For validation, one of the operating surgeons also conducted this assessment blinded. Statistical analysis was conducted using SPSS Statistics Version 22 (IBM, Armonk, New York), with a *p* value of < 0.05 considered to be significant.

Data are presented as: median [interquartile range], unless stated. Times are presented in decimal. For (non-parametric) time and error scores, data were log_10_ transformed, and the one-way ANCOVA was used, including gallbladder grade and consultant as covariates. The independent *t* test or Mann–Whitney *U* test was used to compare continuous parametric or non-parametric data respectively. The Chi square test of two proportions or the Chi square test of homogeneity (*r* × 2) were used to compare data with two categories or more respectively. The Fisher’s exact (2 × 2) or (*r* × 2) tests were used in place of these when minimum sample sizes were not reached.

This trial was registered (NCT02858986) and approved by the Health Research Authority and Oxford B Research Ethics Committee (16/SC/0414).

## Results

**Fig. 2:**
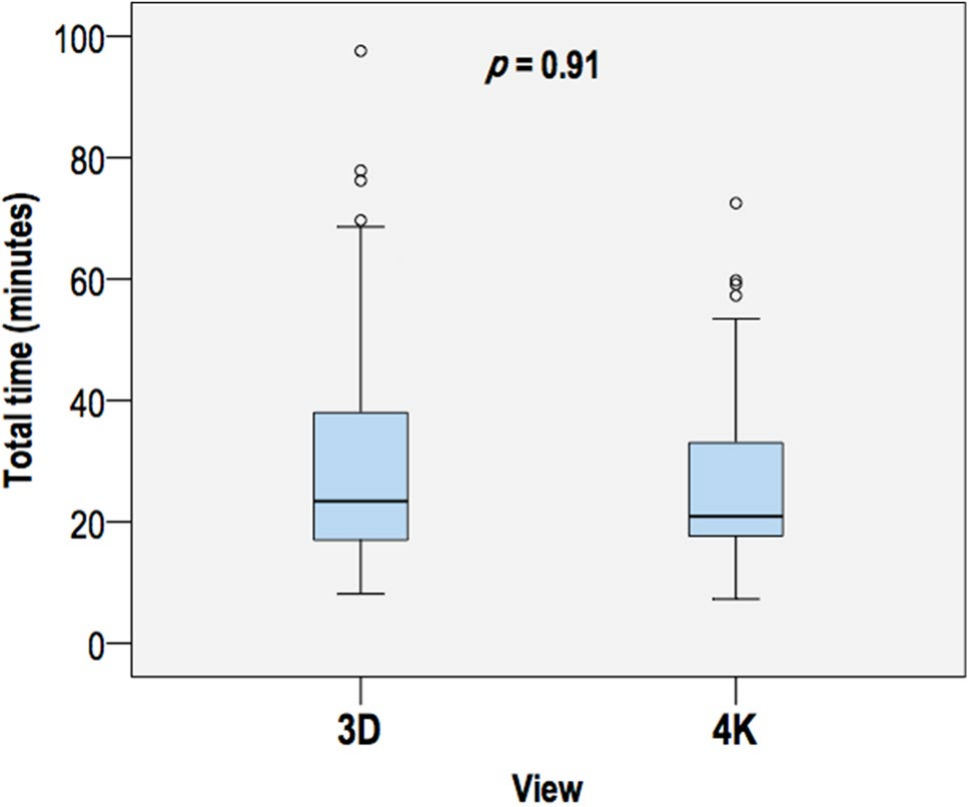
Time to complete laparoscopic cholecystectomy by view. Circles denote values 1.5–3 times the interquartile range

**Fig. 3:**
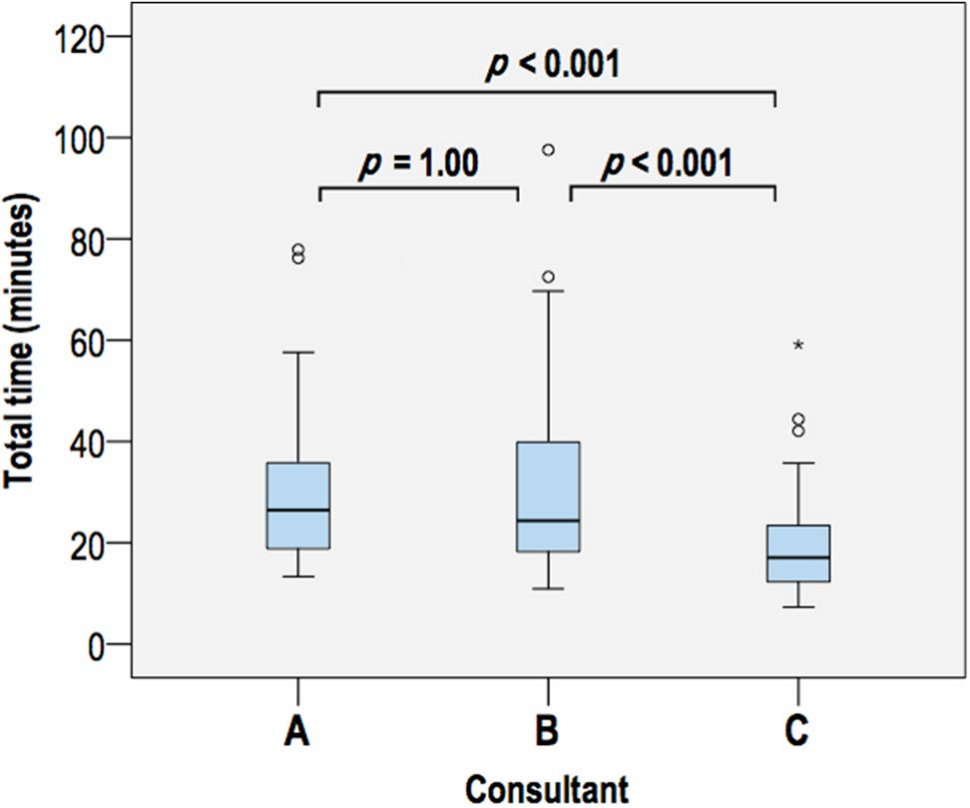
Time to complete laparoscopic cholecystectomy by consultant. Circles denote values 1.5–3 times the interquartile range. Asterisks denote values more than 3 times outside the interquartile range

One hundred and twenty individuals were randomised and *n* = 109 were included for analysis (Fig. 1). There were no differences in preoperative patient characteristics, indications for surgery or gallbladder grade between groups (Table 1). One cholecystectomy was performed during the index admission; this was a grade 3 gallbladder (in the 4K arm). The three consultants performed similar proportions of 3D and 4K cases (Table 2), and suffered no significant side effects during the trial. Twenty on table cholangiograms were performed in each group, plus one (failed) attempt in the 3D group. The operative time was no different between 3D and 4K (23.41 min [17.00–37.98] vs 20.90 min [17.67–33.03]; *p* = 0.91; Fig. 2). The grade of gallbladder strongly affected the time to complete laparoscopic cholecystectomy (*p* < 0.001, partial eta squared = 0.39). This effect was less strong for the operating surgeon (*p* < 0.001, partial eta squared = 0.12; Fig. 3).

**Table 1:** Patient characteristics

	3D	4K	*P* value
Gender			
Male	14	11	0.46
Female	40	44	
Age [mean (SD)]	56.2 (13.4)	55.0 (14.6)	0.66
Indication			
Biliary colic	31	38	
Calculous cholecystitis	14	10	
Obstructive jaundice (gallstones)	6	5	0.63
Polyp	2	1	
Gallstone pancreatitis	1	–	
Cholangitis (gallstones)	–	1	
BMI [median (IQR)]	27.7 (25.6–32.6)	28.7 (23.4–33.7)	0.94
ASA grade			
1	16	18	
2	33	32	0.96
3	5	5	
Gall bladder grade			
1	6	5	
2	31	38	0.44
3	17	12	
Total	54	55	

*ASA* American Society of Anesthesiologists Physical Status Classification System, *BMI* body mass index, *IQR* interquartile range, *SD* standard deviation

**Table 2:** Operations by consultant and view

	3D	4K	Total
Consultant			
A	17	18	35
B	25	21	46
C	12	16	28
Total	54	55	109

**Fig. 4:**
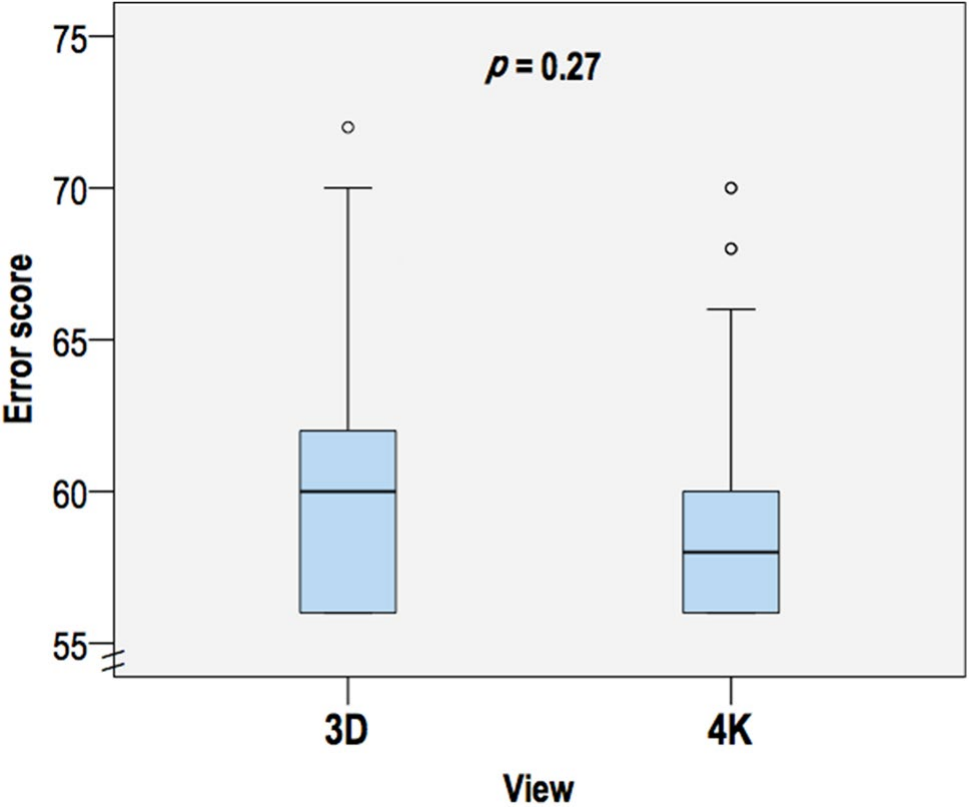
Error score during laparoscopic cholecystectomy by view. Circles denote values 1.5–3 times the interquartile range (note: overlapping outliers—four in total for 4K view)

The error scores were no different between 3D and 4K (60 [56–62] vs 58 [56–60]; *p* = 0.27; Fig. 4), nor between operating surgeons (*p* = 0.24). Only the grade of gallbladder had an effect on the error score (*p* < 0.001, partial eta squared = 0.17). The breakdown of the weighted error scores is available in Supplement 2. There was no difference in the rate of each minor or major error between 3D and 4K, except for gallbladder perforation with stone spillage, which was more common with 3D versus 4K (Tables 3, 4). No significant major errors occurred. The occurrence of any major error, any minor error, or either of a major/minor error was not significantly different between the groups. A Cohen’s kappa coefficient (κ) was > 0.50 for each error, demonstrating a moderate (or better) level of agreement between the two assessors regarding whether an error had occurred or not (dichotomous judgment).

**Table 3:** Minor errors

	3D	4K	*P* value
Injury to gallbladder with bile spilled	19	16	0.50
Liver injury, by diathermy	8	11	0.48
Clip incompletely on cystic artery	7	4	0.32
Clip incompletely on cystic duct	8	4	0.21
Misplaced clip fallen into abdomen	3	2	0.68
Cystic artery or branches not identified initially	3	2	0.68

**Table 4:** Major errors

	3D	4K	*P* value
Gallbladder injury with stones spilled	7	1	0.03
Liver injury with bleeding	8	5	0.36
Unintentional cystic duct division	0	0	N/A
Cystic artery injury	6	6	0.97
Other major vascular injury	0	0	N/A
Duct injury—CBD/right hepatic/Accessory	0	0	N/A
Injury to other abdominal viscus	0	0	N/A

No major complications occurred, and there were no differences in reattendance and complication rates between 3D and 4K (Table 5).

**Table 5:** 30-day complications and reattendances

	3D	4K	*P* value
Postoperative pain (reattendance)	3	2	0.68
Intraoperative dysrhythmia	1	1	1.00
Infected umbilical wound	1	0	0.50
Concern about wound ooze (reattendance)	0	1	1.00
Urinary retention	1	1	1.00
Upper gastrointestinal bleed on dalteparin	1	0	0.50
Constipation (reattendance)	1	0	0.50

Analysis of grade 3 gallbladder operations (the most difficult procedures), showed no difference between 3D and 4K for time to complete the procedure (48.00 min [34.97–68.60] vs 38.69 min [28.51–47.87]; *p* = 0.15) or error score (64 [62–64] vs 60 [56–65]; *p* = 0.33). Neither was there a difference between surgeons for these outcomes.

## Discussion

This is the first randomised-controlled trial in a clinical setting to determine the utility of 3D high-definition imaging against a 4K system. We found that the binocular vision provided by a 3D HD laparoscopic system does not reduce operative time, intraoperative error score, 30-day complication rate or reattendance following laparoscopic cholecystectomy, when compared to the monocular vision provided by a 4K system.

Harada et al. compared 3D HD and 4K laparoscopes in a simulator environment [19], and reported that performance in knotting and suturing tasks was equivalent for 3D and 4K when performed in confined spaces; however, 3D was superior to 4K in more open spaces. This difference was attributed to the shadows in confined spaces, and hence the enhanced monocular depth cues. The present study is the first clinical study to compare 3D and 4K technologies. Of the three previous studies among expert surgeons comparing 3D HD with 2D HD laparoscopic cholecystectomy, Sahu et al. reported a 14 min (26%) time benefit with 3D [9], Bilgen et al. reported a 9 min (31%) benefit with 3D [20], whilst Tung et al. reported no difference [21]. However, these were smaller studies with less statistical power. Koppatz et al. recently reported the largest 3D HD versus 2D HD laparoscopic cholecystectomy study to date, with over 100 participants in each arm [22]. They showed no difference in operative times or complication rates between 3D and 2D, including on subgroup analysis for surgical experience. As in the current study, these trials demonstrated no major complications with either the 3D or 2D technologies. We found the only difference in errors between 3D and 4K was more frequent gallbladder perforation with stone spillage in the 3D group. It has been suggested that 4K imaging may provide greater anatomical discrimination than HD [23]. This might improve dissection, and explain the lower rate of stone spillage in the 4K group. However, if this were the case, it would also be expected that bile spillage rates would also be less. Nonetheless, this supports the recent suggestion that specimen quality may be a useful outcome measure in future 3D versus 2D laparoscopic trials [24].

Data from our institution have suggested that 3D HD has a time advantage over 2D HD in higher-grade gallbladder operations, and may reduce the number of errors [25]. In the current study, no difference was found between 3D HD and 4K in grade 3 gallbladder operations.

The limitations to this study were that the primary outcome was measured unblinded, and it was not possible to power for subgroup analysis. However, in view of the objective nature of time measurement, this was accepted. Measuring surgical performance is difficult, and time and error outcomes may be difficult to power, and may not relate to clinical outcomes. The operative times proved to be shorter than anticipated, which affects the power of the study and increases the risk of type II error. This was due to the fact that only the laparoscopic portion of the operation was analysed. However, we feel that these operative times were not exceptional. Another recent study in our centre, using similar methods to the current trial, and with similar operative times, showed no difference in the time to complete 3D HD and 2D HD laparoscopic cholecystectomy [25]. We controlled for grade of surgeon by limiting participation to Consultant surgeons only. Randomisation by Consultant surgeon was not performed a priori as we had not anticipated a significant time difference between Consultant surgeons. Finally, this study was of 3D HD and 4K technologies only. A clinical comparison between 2D HD and 4K technologies would be beneficial in future studies.

Laparoscopic cholecystectomy, as a relatively simple procedure, may not reveal a difference between 3D and 4K technologies. Pooled data from trials including more complicated general surgical procedures have shown that operative time could be reduced with 3D systems when compared to 2D HD systems. However, only a 4% decrease in operative time was demonstrated [12]. Delphi processes and pilot studies are required to develop new tools for assessing 3D, 2D and 4K technologies. Performance assessment in laparoscopic surgery has been highlighted as a research priority by EAES [26]. Furthermore, this trial included Consultant Surgeons only. Future studies should investigate trainees’ performance with 3D and 4K systems.

This study suggests that a 3D HD system does not reduce operative time or error scores during laparoscopic cholecystectomy when compared to a 4K system. Variability between surgeons has a demonstrable effect on operative performance, as does gallbladder grade, and these should be considered when planning similar clinical trials.

Provisional results from this study have been presented at the International Congress of the EAES [27, 28].

## Electronic supplementary material

Below is the link to the electronic supplementary material.

Supplementary material 1 Scoring chart for the technical skills checklist, following Sarker et al. [17] (TIFF 139 kb)

Supplementary material 2 Breakdown of weighted error scores (DOCX 186 kb)

## References

[1] Nguyen KT, Marsh JW, Tsung A, Steel JJL, Gamblin TC, Geller DA (2011). Comparative benefits of laparoscopic vs open hepatic resection. Arch Surg.

[2] Veldkamp R, Kuhry E, Hop WCJ, Jeekel J, Kazemier G, Bonjer HJ, Haglind E, Påhlman L, Cuesta MA, Msika S, Morino M, Lacy AM (2005). Laparoscopic surgery versus open surgery for colon cancer: short-term outcomes of a randomised trial. Lancet Oncol.

[3] Obermair A, Manolitsas TP, Leung Y, Hammond IG, McCartney AJ (2005). Total laparoscopic hysterectomy versus total abdominal hysterectomy for obese women with endometrial cancer. Int J Gynecol Cancer.

[4] Ballantyne GH (2002). The pitfalls of laparoscopic surgery: challenges for robotics and telerobotic surgery. Surg Laparosc Endosc Percutan Tech.

[5] Ziogas IA, Tsoulfas G (2017). Advances and challenges in laparoscopic surgery in the management of hepatocellular carcinoma. World J Gastrointest Surg.

[6] Schwab K, Smith R, Brown V, Whyte M, Jourdan I (2017). Evolution of stereoscopic imaging in surgery and recent advances. World J Gastrointest Endosc.

[7] Ohuchida K, Eishi N, Ieiri S, Tomohiko A, Tetsuo I, Tanaka M, Hashizume M (2013). New Advances in Three-Dimensional Endoscopic Surgery. J Gastrointest Dig Syst.

[8] Abel E, Fotiadis N, Miah M, White P (2015). Defining optical distortion in rigid endoscopes. Laryngoscope.

[9] Sahu D, Mathew MJ, Reddy PK (2014). 3D Laparoscopy—help or Hype; initial experience of a tertiary health centre. J Clin Diagn Res.

[10] Su H, Li J, Zhang H, Li J, Wang S (2016). Using motion parallax for laparoscopic surgery. Int J Med Robot Comput Assist Surg.

[11] Sørensen SMD, Savran MM, Konge L, Bjerrum F (2016). Three-dimensional versus two-dimensional vision in laparoscopy: a systematic review. Surg Endosc.

[12] Arezzo A, Vettoretto N, Francis NK, Bonino MA, Curtis NJ, Amparore D, Arolfo S, Barberio M, Boni L, Brodie R, Bouvy N, Cassinotti E, Carus T, Checcucci E, Custers P, Diana M, Jansen M, Jaspers J, Marom G (2018). The use of 3D laparoscopic imaging systems in surgery: EAES consensus development conference 2018. Surg Endosc.

[13] Singh A, Saraiya R (2013). Three-dimensional endoscopy in sinus surgery. Curr Opin Otolaryngol Head Neck Surg.

[14] Fergo C, Burcharth J, Pommergaard H-C, Rosenberg J (2016). Age is highly associated with stereo blindness among surgeons: a cross-sectional study. Surg Endosc.

[15] Lai ECH, Yang GPC, Tang CN, Yih PCL, Chan OCY, Li MKW (2011). Prospective randomized comparative study of single incision laparoscopic cholecystectomy versus conventional four-port laparoscopic cholecystectomy. Am J Surg.

[16] Pisanu A, Reccia I, Porceddu G, Uccheddu A (2012). Meta-analysis of Prospective Randomized Studies Comparing Single-Incision Laparoscopic Cholecystectomy (SILC) and Conventional Multiport Laparoscopic Cholecystectomy (CMLC). J Gastrointest Surg.

[17] Sarker SK, Chang A, Vincent C (2006). Technical and technological skills assessment in laparoscopic surgery. JSLS.

[18] Hanna GB, Shimi SM, Cuschieri A (1998). Randomised study of influence of two-dimensional versus three-dimensional imaging on performance of laparoscopic cholecystectomy. Lancet.

[19] Harada H, Kanaji S, Hasegawa H, Yamamoto M, Matsuda Y, Yamashita K, Matsuda T, Oshikiri T, Sumi Y, Nakamura T, Suzuki S, Kakeji Y (2018). The effect on surgical skills of expert surgeons using 3D/HD and 2D/4K resolution monitors in laparoscopic phantom tasks. Surg Endosc.

[20] Bilgen K, Ustün M, Karakahya M, Işik S, Sengül S, Cetinkünar S, Küçükpinar TH (2013). Comparison of 3D imaging and 2D imaging for performance time of laparoscopic cholecystectomy. Surg Laparosc Endosc Percutan Tech.

[21] Tung KL, Yang GP, Li MK (2015). Comparative study of 2-D and bichanneled 3-D laparoscopic images: is there a difference?. Asian J Endosc Surg.

[22] Koppatz H, Harju J, Sirén J, Mentula P, Scheinin T, Sallinen V (2019). Three-dimensional versus two-dimensional high-definition laparoscopy in cholecystectomy: a prospective randomized controlled study. Surg Endosc.

[23] Rigante M, La Rocca G, Lauretti L, D’Alessandris GQ, Mangiola A, Anile C, Olivi A, Paludetti G (2017). Preliminary experience with 4K ultra-high definition endoscope: analysis of pros and cons in skull base surgery. Acta Otorhinolaryngol Ital.

[24] Curtis NJ, Conti JA, Dalton R, Rockall TA, Allison AS, Ockrim JB, Jourdan IC, Torkington J, Phillips S, Allison J, Hanna GB, Francis NK (2019). 2D versus 3D laparoscopic total mesorectal excision: a developmental multicentre randomised controlled trial. Surg Endosc.

[25] Schwab K (2017) Does three-dimensional technology transfer from the laboratory to the operating theatre with benefits to surgical efficiency and patient safety? Master’s Thesis. http://epubs.surrey.ac.uk/845785/1/Thesis%20after%20corrections.pdf. Accessed 1 Mar 2019

[26] Francis N, Kazaryan AM, Pietrabissa A, Goitein D, Yiannakopoulou E, Agresta F, Khatkov I, Schulze S, Arulampalam T, Tomulescu V, Kim Y-W, Targarona EM, Zaninotto G (2017). A research agenda for the European Association for Endoscopic Surgeons (EAES). Surg Endosc.

[27] Dunstan M, Smith R, Schwab K, Whyte M, Rockall T, Jourdan I (2017). Does a 3D laparoscope reduce the time to perform cholecystectomy when compared to a 4K laparoscope? A randomised controlled trial. In: 25th International Congress of the European Association for Endoscopic Surgery (EAES), Frankfurt, Germany, 14–17 June 2017: Oral Presentations. Surg Endosc.

[28] Dunstan M, Smith R, Schwab K, Scala A, Gatenby P, Whyte M, Rockall T, Jourdan I (2018). 3D versus 4K laparoscopic cholecystectomy: A randomised controlled trial. In: 26th International Congress of the European Association for Endoscopic Surgery (EAES), London, United Kingdom, 30 May–1 June 2018: Oral Presentations. Surg Endosc.

