# Supplementary figures and images for: Is 3D faster and safer than 4K laparoscopic cholecystectomy? A randomised-controlled trial
Source: Surg Endosc. 2019 Jul 18;34(4):1729–35. doi: 10.1007/s00464-019-06958-w (PMC7093366; doi:10.1007/s00464-019-06958-w)

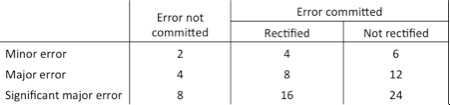

Supplement: Supplementary file 1 — Supplementary material 1 Scoring chart for the technical skills checklist, following Sarker et al. [17] (TIFF 139 kb) [file 464_2019_6958_MOESM1_ESM.tif]
