# Supplementary material for: Is 3D faster and safer than 4K laparoscopic cholecystectomy? A randomised-controlled trial
Source: Surg Endosc. 2019 Jul 18;34(4):1729–35. doi: 10.1007/s00464-019-06958-w (PMC7093366; doi:10.1007/s00464-019-06958-w)
Supplement: Supplementary file 2 — Supplementary material 2 Breakdown of weighted error scores (DOCX 186 kb) [file 464_2019_6958_MOESM2_ESM.docx]

*Supplement 2 – Breakdown of weighted error scores*

|  |  |  |  | **Minor errors** | | | | | | **Major errors** | | | | | **Significant major errors** | | |  |
| --- | --- | --- | --- | --- | --- | --- | --- | --- | --- | --- | --- | --- | --- | --- | --- | --- | --- | --- |
| **Pt.** | **View** | **Cons.** | **Grade** | **Injury to gallbladder with bile spilled** | **Liver injury, by diathermy** | **Clip incompletely on cystic artery** | **Clip incompletely on cystic duct** | **Misplaced clip fallen into abdomen** | **Cystic artery or branches not identified initially** | **Gallbladder injury with stones spilled** | **Liver injury with bleeding** | **Unintentional cystic duct division** | **Cystic artery injury** | **Loss of pneumoperitoneum due to surgery** | **Other major vascular injury** | **Duct injury - CBD/ Right hepatic/ Accessory** | **Injury to other abdominal viscus** | **Error score:** |
| 1 | 3D | A | 3 | Error rectified - 4 | Error rectified - 4 | No error - 2 | No error - 2 | No error - 2 | No error - 2 | Error rectified - 8 | Error rectified - 8 | No error - 4 | Error rectified - 8 | No error - 4 | No error - 8 | No error - 8 | No error - 8 | 72 |
| 2 | 4K | A | 2 | No error - 2 | No error - 2 | No error - 2 | No error - 2 | No error - 2 | No error - 2 | No error - 4 | No error - 4 | No error - 4 | No error - 4 | No error - 4 | No error - 8 | No error - 8 | No error - 8 | 56 |
| 3 | 3D | B | 1 | No error - 2 | No error - 2 | No error - 2 | No error - 2 | No error - 2 | No error - 2 | No error - 4 | No error - 4 | No error - 4 | No error - 4 | No error - 4 | No error - 8 | No error - 8 | No error - 8 | 56 |
| 4 | 4K | B | 3 | Error rectified - 4 | No error - 2 | No error - 2 | No error - 2 | No error - 2 | No error - 2 | No error - 4 | No error - 4 | No error - 4 | No error - 4 | No error - 4 | No error - 8 | No error - 8 | No error - 8 | 58 |
| 5 | 4K | B | 2 | No error - 2 | No error - 2 | Error rectified - 4 | No error - 2 | No error - 2 | No error - 2 | No error - 4 | No error - 4 | No error - 4 | No error - 4 | No error - 4 | No error - 8 | No error - 8 | No error - 8 | 58 |
| 6 | 4K | B | 1 | No error - 2 | Error not rectified - 6 | No error - 2 | No error - 2 | No error - 2 | No error - 2 | No error - 4 | No error - 4 | No error - 4 | No error - 4 | No error - 4 | No error - 8 | No error - 8 | No error - 8 | 60 |
| 7 | 3D | A | 2 | No error - 2 | No error - 2 | No error - 2 | No error - 2 | No error - 2 | Error rectified - 4 | No error - 4 | No error - 4 | No error - 4 | No error - 4 | No error - 4 | No error - 8 | No error - 8 | No error - 8 | 58 |
| 8 | 3D | A | 3 | Error rectified - 4 | Error rectified - 4 | No error - 2 | No error - 2 | No error - 2 | No error - 2 | Error rectified - 8 | No error - 4 | No error - 4 | No error - 4 | No error - 4 | No error - 8 | No error - 8 | No error - 8 | 64 |
| 9 | 3D | A | 2 | No error - 2 | No error - 2 | No error - 2 | No error - 2 | No error - 2 | No error - 2 | No error - 4 | No error - 4 | No error - 4 | No error - 4 | No error - 4 | No error - 8 | No error - 8 | No error - 8 | 56 |
| 10 | 4K | C | 2 | No error - 2 | No error - 2 | No error - 2 | No error - 2 | No error - 2 | No error - 2 | No error - 4 | No error - 4 | No error - 4 | No error - 4 | No error - 4 | No error - 8 | No error - 8 | No error - 8 | 56 |
| 11 | 4K | C | 1 | No error - 2 | No error - 2 | No error - 2 | No error - 2 | No error - 2 | No error - 2 | No error - 4 | No error - 4 | No error - 4 | No error - 4 | No error - 4 | No error - 8 | No error - 8 | No error - 8 | 56 |
| 12 | 3D | A | 1 | No error - 2 | No error - 2 | No error - 2 | No error - 2 | No error - 2 | No error - 2 | No error - 4 | No error - 4 | No error - 4 | No error - 4 | No error - 4 | No error - 8 | No error - 8 | No error - 8 | 56 |
| 13 | 4K | A | 2 | No error - 2 | No error - 2 | No error - 2 | No error - 2 | No error - 2 | No error - 2 | No error - 4 | No error - 4 | No error - 4 | No error - 4 | No error - 4 | No error - 8 | No error - 8 | No error - 8 | 56 |
| 14 | 4K | A | 2 | No error - 2 | No error - 2 | No error - 2 | No error - 2 | No error - 2 | No error - 2 | No error - 4 | No error - 4 | No error - 4 | Error rectified - 8 | No error - 4 | No error - 8 | No error - 8 | No error - 8 | 60 |
| 15 | 3D | B | 2 | No error - 2 | No error - 2 | No error - 2 | Error rectified - 4 | No error - 2 | No error - 2 | No error - 4 | Error rectified - 8 | No error - 4 | No error - 4 | No error - 4 | No error - 8 | No error - 8 | No error - 8 | 62 |
| 16 | 3D | B | 3 | No error - 2 | No error - 2 | No error - 2 | No error - 2 | No error - 2 | No error - 2 | No error - 4 | No error - 4 | No error - 4 | No error - 4 | No error - 4 | No error - 8 | No error - 8 | No error - 8 | 56 |
| 17 | 4K | B | 3 | Error rectified - 4 | No error - 2 | No error - 2 | No error - 2 | No error - 2 | No error - 2 | No error - 4 | No error - 4 | No error - 4 | Error rectified - 8 | No error - 4 | No error - 8 | No error - 8 | No error - 8 | 62 |
| 18 | 4K | B | 2 | No error - 2 | No error - 2 | No error - 2 | No error - 2 | No error - 2 | No error - 2 | No error - 4 | No error - 4 | No error - 4 | No error - 4 | No error - 4 | No error - 8 | No error - 8 | No error - 8 | 56 |
| 19 | 4K | A | 3 | No error - 2 | Error not rectified - 6 | No error - 2 | Error rectified - 4 | No error - 2 | No error - 2 | No error - 4 | Error rectified - 8 | No error - 4 | Error rectified - 8 | No error - 4 | No error - 8 | No error - 8 | No error - 8 | 70 |
| 20 | 3D | C | 2 | No error - 2 | No error - 2 | No error - 2 | No error - 2 | No error - 2 | No error - 2 | No error - 4 | No error - 4 | No error - 4 | No error - 4 | No error - 4 | No error - 8 | No error - 8 | No error - 8 | 56 |
| 21 | 3D | A | 1 | No error - 2 | No error - 2 | No error - 2 | No error - 2 | No error - 2 | No error - 2 | No error - 4 | No error - 4 | No error - 4 | No error - 4 | No error - 4 | No error - 8 | No error - 8 | No error - 8 | 56 |
| 22 | 3D | A | 2 | Error rectified - 4 | No error - 2 | No error - 2 | No error - 2 | No error - 2 | No error - 2 | No error - 4 | Error rectified - 8 | No error - 4 | No error - 4 | No error - 4 | No error - 8 | No error - 8 | No error - 8 | 62 |
| 23 | 4K | A | 1 | No error - 2 | No error - 2 | No error - 2 | No error - 2 | No error - 2 | No error - 2 | No error - 4 | No error - 4 | No error - 4 | No error - 4 | No error - 4 | No error - 8 | No error - 8 | No error - 8 | 56 |
| 24 | 4K | B | 2 | No error - 2 | No error - 2 | Error rectified - 4 | No error - 2 | Error rectified - 4 | No error - 2 | No error - 4 | No error - 4 | No error - 4 | No error - 4 | No error - 4 | No error - 8 | No error - 8 | No error - 8 | 60 |
| 25 | 3D | B | 3 | Error not rectified - 6 | No error - 2 | No error - 2 | No error - 2 | No error - 2 | Error rectified - 4 | No error - 4 | No error - 4 | No error - 4 | No error - 4 | No error - 4 | No error - 8 | No error - 8 | No error - 8 | 62 |
| 26 | 4K | B | 2 | Error not rectified - 6 | Error not rectified - 6 | No error - 2 | No error - 2 | No error - 2 | No error - 2 | No error - 4 | Error rectified - 8 | No error - 4 | No error - 4 | No error - 4 | No error - 8 | No error - 8 | No error - 8 | 68 |
| 27 | 4K | B | 2 | No error - 2 | No error - 2 | No error - 2 | No error - 2 | No error - 2 | No error - 2 | No error - 4 | No error - 4 | No error - 4 | No error - 4 | No error - 4 | No error - 8 | No error - 8 | No error - 8 | 56 |
| 28 | 3D | A | 3 | No error - 2 | No error - 2 | Error rectified - 4 | No error - 2 | Error rectified - 4 | No error - 2 | Error rectified - 8 | Error rectified - 8 | No error - 4 | No error - 4 | No error - 4 | No error - 8 | No error - 8 | No error - 8 | 68 |
| 29 | 3D | A | 2 | No error - 2 | No error - 2 | No error - 2 | No error - 2 | No error - 2 | No error - 2 | No error - 4 | No error - 4 | No error - 4 | No error - 4 | No error - 4 | No error - 8 | No error - 8 | No error - 8 | 56 |
| 30 | 3D | C | 3 | Error not rectified - 6 | No error - 2 | No error - 2 | Error rectified - 4 | Error not rectified - 6 | No error - 2 | No error - 4 | Error rectified - 8 | No error - 4 | No error - 4 | No error - 4 | No error - 8 | No error - 8 | No error - 8 | 70 |
| 31 | 3D | B | 2 | No error - 2 | No error - 2 | No error - 2 | No error - 2 | No error - 2 | No error - 2 | No error - 4 | No error - 4 | No error - 4 | No error - 4 | No error - 4 | No error - 8 | No error - 8 | No error - 8 | 56 |
| 32 | 4K | A | 2 | Error not rectified - 6 | No error - 2 | No error - 2 | No error - 2 | No error - 2 | No error - 2 | No error - 4 | No error - 4 | No error - 4 | No error - 4 | No error - 4 | No error - 8 | No error - 8 | No error - 8 | 60 |
| 33 | 3D | A | 3 | Error not rectified - 6 | Error not rectified - 6 | No error - 2 | No error - 2 | No error - 2 | No error - 2 | No error - 4 | Error rectified - 8 | No error - 4 | No error - 4 | No error - 4 | No error - 8 | No error - 8 | No error - 8 | 68 |
| 34 | 3D | B | 2 | No error - 2 | No error - 2 | No error - 2 | No error - 2 | No error - 2 | No error - 2 | No error - 4 | No error - 4 | No error - 4 | No error - 4 | No error - 4 | No error - 8 | No error - 8 | No error - 8 | 56 |
| 35 | 4K | B | 2 | No error - 2 | No error - 2 | No error - 2 | No error - 2 | No error - 2 | No error - 2 | No error - 4 | No error - 4 | No error - 4 | No error - 4 | No error - 4 | No error - 8 | No error - 8 | No error - 8 | 56 |
| 36 | 4K | B | 2 | No error - 2 | No error - 2 | No error - 2 | No error - 2 | No error - 2 | No error - 2 | No error - 4 | Error rectified - 8 | No error - 4 | No error - 4 | No error - 4 | No error - 8 | No error - 8 | No error - 8 | 60 |
| 37 | 4K | B | 2 | No error - 2 | Error not rectified - 6 | No error - 2 | No error - 2 | No error - 2 | No error - 2 | No error - 4 | No error - 4 | No error - 4 | No error - 4 | No error - 4 | No error - 8 | No error - 8 | No error - 8 | 60 |
| 38 | 4K | C | 2 | No error - 2 | No error - 2 | No error - 2 | No error - 2 | No error - 2 | No error - 2 | No error - 4 | No error - 4 | No error - 4 | No error - 4 | No error - 4 | No error - 8 | No error - 8 | No error - 8 | 56 |
| 39 | 3D | B | 2 | No error - 2 | No error - 2 | No error - 2 | No error - 2 | No error - 2 | No error - 2 | No error - 4 | No error - 4 | No error - 4 | No error - 4 | No error - 4 | No error - 8 | No error - 8 | No error - 8 | 56 |
| 40 | 4K | B | 2 | No error - 2 | No error - 2 | No error - 2 | No error - 2 | No error - 2 | No error - 2 | No error - 4 | No error - 4 | No error - 4 | No error - 4 | No error - 4 | No error - 8 | No error - 8 | No error - 8 | 56 |
| 41 | 4K | B | 2 | No error - 2 | No error - 2 | No error - 2 | Error rectified - 4 | No error - 2 | No error - 2 | No error - 4 | No error - 4 | No error - 4 | No error - 4 | No error - 4 | No error - 8 | No error - 8 | No error - 8 | 58 |
| 42 | 3D | B | 2 | Error not rectified - 6 | Error not rectified - 6 | Error rectified - 4 | No error - 2 | No error - 2 | No error - 2 | No error - 4 | No error - 4 | No error - 4 | Error rectified - 8 | No error - 4 | No error - 8 | No error - 8 | No error - 8 | 70 |
| 43 | 3D | B | 3 | No error - 2 | No error - 2 | No error - 2 | No error - 2 | No error - 2 | No error - 2 | Error rectified - 8 | No error - 4 | No error - 4 | Error rectified - 8 | No error - 4 | No error - 8 | No error - 8 | No error - 8 | 64 |
| 44 | 3D | A | 3 | No error - 2 | Error not rectified - 6 | No error - 2 | No error - 2 | No error - 2 | No error - 2 | No error - 4 | Error rectified - 8 | No error - 4 | No error - 4 | No error - 4 | No error - 8 | No error - 8 | No error - 8 | 64 |
| 45 | 3D | A | 2 | No error - 2 | No error - 2 | No error - 2 | No error - 2 | No error - 2 | No error - 2 | No error - 4 | No error - 4 | No error - 4 | No error - 4 | No error - 4 | No error - 8 | No error - 8 | No error - 8 | 56 |
| 46 | 4K | B | 2 | Error rectified - 4 | No error - 2 | No error - 2 | No error - 2 | No error - 2 | No error - 2 | No error - 4 | No error - 4 | No error - 4 | No error - 4 | No error - 4 | No error - 8 | No error - 8 | No error - 8 | 58 |
| 47 | 4K | C | 2 | No error - 2 | No error - 2 | No error - 2 | No error - 2 | No error - 2 | No error - 2 | No error - 4 | No error - 4 | No error - 4 | No error - 4 | No error - 4 | No error - 8 | No error - 8 | No error - 8 | 56 |
| 48 | 4K | B | 2 | No error - 2 | No error - 2 | No error - 2 | No error - 2 | No error - 2 | No error - 2 | No error - 4 | No error - 4 | No error - 4 | No error - 4 | No error - 4 | No error - 8 | No error - 8 | No error - 8 | 56 |
| 49 | 4K | B | 2 | No error - 2 | No error - 2 | No error - 2 | No error - 2 | No error - 2 | No error - 2 | No error - 4 | No error - 4 | No error - 4 | No error - 4 | No error - 4 | No error - 8 | No error - 8 | No error - 8 | 56 |
| 50 | 3D | B | 1 | No error - 2 | No error - 2 | No error - 2 | No error - 2 | No error - 2 | No error - 2 | No error - 4 | No error - 4 | No error - 4 | No error - 4 | No error - 4 | No error - 8 | No error - 8 | No error - 8 | 56 |
| 51 | 4K | A | 2 | No error - 2 | No error - 2 | No error - 2 | No error - 2 | No error - 2 | No error - 2 | No error - 4 | No error - 4 | No error - 4 | No error - 4 | No error - 4 | No error - 8 | No error - 8 | No error - 8 | 56 |
| 52 | 4K | C | 2 | No error - 2 | No error - 2 | Error rectified - 4 | No error - 2 | No error - 2 | No error - 2 | No error - 4 | No error - 4 | No error - 4 | No error - 4 | No error - 4 | No error - 8 | No error - 8 | No error - 8 | 58 |
| 53 | 4K | C | 2 | No error - 2 | No error - 2 | No error - 2 | No error - 2 | No error - 2 | No error - 2 | No error - 4 | No error - 4 | No error - 4 | No error - 4 | No error - 4 | No error - 8 | No error - 8 | No error - 8 | 56 |
| 54 | 3D | C | 2 | Error not rectified - 6 | No error - 2 | Error rectified - 4 | No error - 2 | No error - 2 | No error - 2 | No error - 4 | No error - 4 | No error - 4 | No error - 4 | No error - 4 | No error - 8 | No error - 8 | No error - 8 | 62 |
| 55 | 3D | C | 2 | No error - 2 | No error - 2 | No error - 2 | No error - 2 | No error - 2 | No error - 2 | No error - 4 | No error - 4 | No error - 4 | No error - 4 | No error - 4 | No error - 8 | No error - 8 | No error - 8 | 56 |
| 56 | 3D | B | 1 | No error - 2 | Error not rectified - 6 | No error - 2 | No error - 2 | No error - 2 | No error - 2 | No error - 4 | No error - 4 | No error - 4 | No error - 4 | No error - 4 | No error - 8 | No error - 8 | No error - 8 | 60 |
| 57 | 4K | C | 3 | Error rectified - 4 | Error not rectified - 6 | No error - 2 | Error rectified - 4 | No error - 2 | Error rectified - 4 | Error rectified - 8 | No error - 4 | No error - 4 | No error - 4 | No error - 4 | No error - 8 | No error - 8 | No error - 8 | 70 |
| 58 | 4K | C | 2 | No error - 2 | No error - 2 | No error - 2 | No error - 2 | No error - 2 | No error - 2 | No error - 4 | No error - 4 | No error - 4 | No error - 4 | No error - 4 | No error - 8 | No error - 8 | No error - 8 | 56 |
| 59 | 3D | B | 1 | No error - 2 | No error - 2 | No error - 2 | No error - 2 | No error - 2 | No error - 2 | No error - 4 | No error - 4 | No error - 4 | No error - 4 | No error - 4 | No error - 8 | No error - 8 | No error - 8 | 56 |
| 60 | 4K | A | 1 | No error - 2 | No error - 2 | No error - 2 | No error - 2 | No error - 2 | No error - 2 | No error - 4 | No error - 4 | No error - 4 | No error - 4 | No error - 4 | No error - 8 | No error - 8 | No error - 8 | 56 |
| 61 | 4K | A | 2 | Error rectified - 4 | No error - 2 | No error - 2 | No error - 2 | No error - 2 | No error - 2 | No error - 4 | No error - 4 | No error - 4 | No error - 4 | No error - 4 | No error - 8 | No error - 8 | No error - 8 | 58 |
| 62 | 3D | B | 2 | No error - 2 | No error - 2 | No error - 2 | No error - 2 | No error - 2 | No error - 2 | No error - 4 | No error - 4 | No error - 4 | No error - 4 | No error - 4 | No error - 8 | No error - 8 | No error - 8 | 56 |
| 63 | 3D | B | 2 | No error - 2 | No error - 2 | No error - 2 | No error - 2 | No error - 2 | No error - 2 | No error - 4 | No error - 4 | No error - 4 | No error - 4 | No error - 4 | No error - 8 | No error - 8 | No error - 8 | 56 |
| 64 | 3D | B | 2 | Error not rectified - 6 | No error - 2 | No error - 2 | No error - 2 | No error - 2 | No error - 2 | No error - 4 | No error - 4 | No error - 4 | No error - 4 | No error - 4 | No error - 8 | No error - 8 | No error - 8 | 60 |
| 65 | 3D | B | 3 | Error not rectified - 6 | Error not rectified - 6 | No error - 2 | No error - 2 | No error - 2 | No error - 2 | No error - 4 | No error - 4 | No error - 4 | No error - 4 | No error - 4 | No error - 8 | No error - 8 | No error - 8 | 64 |
| 66 | 4K | C | 2 | Error not rectified - 6 | Error not rectified - 6 | No error - 2 | No error - 2 | No error - 2 | No error - 2 | No error - 4 | No error - 4 | No error - 4 | Error rectified - 8 | No error - 4 | No error - 8 | No error - 8 | No error - 8 | 68 |
| 67 | 4K | C | 3 | Error not rectified - 6 | No error - 2 | No error - 2 | Error rectified - 4 | No error - 2 | No error - 2 | No error - 4 | Error rectified - 8 | No error - 4 | No error - 4 | No error - 4 | No error - 8 | No error - 8 | No error - 8 | 66 |
| 68 | 4K | C | 2 | No error - 2 | No error - 2 | No error - 2 | No error - 2 | Error rectified - 4 | No error - 2 | No error - 4 | Error rectified - 8 | No error - 4 | No error - 4 | No error - 4 | No error - 8 | No error - 8 | No error - 8 | 62 |
| 69 | 3D | C | 3 | No error - 2 | No error - 2 | No error - 2 | No error - 2 | No error - 2 | No error - 2 | No error - 4 | No error - 4 | No error - 4 | No error - 4 | No error - 4 | No error - 8 | No error - 8 | No error - 8 | 56 |
| 70 | 4K | C | 2 | No error - 2 | Error not rectified - 6 | No error - 2 | No error - 2 | No error - 2 | No error - 2 | No error - 4 | No error - 4 | No error - 4 | No error - 4 | No error - 4 | No error - 8 | No error - 8 | No error - 8 | 60 |
| 71 | 4K | C | 2 | No error - 2 | No error - 2 | No error - 2 | No error - 2 | No error - 2 | No error - 2 | No error - 4 | No error - 4 | No error - 4 | No error - 4 | No error - 4 | No error - 8 | No error - 8 | No error - 8 | 56 |
| 72 | 3D | C | 2 | No error - 2 | No error - 2 | No error - 2 | No error - 2 | No error - 2 | No error - 2 | No error - 4 | No error - 4 | No error - 4 | No error - 4 | No error - 4 | No error - 8 | No error - 8 | No error - 8 | 56 |
| 73 | 3D | C | 2 | Error not rectified - 6 | No error - 2 | Error rectified - 4 | Error not rectified - 6 | No error - 2 | No error - 2 | No error - 4 | No error - 4 | No error - 4 | No error - 4 | No error - 4 | No error - 8 | No error - 8 | No error - 8 | 66 |
| 74 | 3D | B | 2 | No error - 2 | Error not rectified - 6 | Error rectified - 4 | No error - 2 | No error - 2 | Error rectified - 4 | No error - 4 | No error - 4 | No error - 4 | No error - 4 | No error - 4 | No error - 8 | No error - 8 | No error - 8 | 64 |
| 75 | 4K | B | 2 | Error not rectified - 6 | Error not rectified - 6 | No error - 2 | No error - 2 | No error - 2 | No error - 2 | No error - 4 | No error - 4 | No error - 4 | No error - 4 | No error - 4 | No error - 8 | No error - 8 | No error - 8 | 64 |
| 76 | 3D | B | 2 | No error - 2 | No error - 2 | No error - 2 | No error - 2 | No error - 2 | No error - 2 | No error - 4 | No error - 4 | No error - 4 | No error - 4 | No error - 4 | No error - 8 | No error - 8 | No error - 8 | 56 |
| 77 | 4K | C | 3 | Error not rectified - 6 | No error - 2 | No error - 2 | No error - 2 | No error - 2 | No error - 2 | No error - 4 | No error - 4 | No error - 4 | No error - 4 | No error - 4 | No error - 8 | No error - 8 | No error - 8 | 60 |
| 78 | 3D | C | 3 | Error not rectified - 6 | No error - 2 | No error - 2 | No error - 2 | No error - 2 | No error - 2 | No error - 4 | No error - 4 | No error - 4 | Error rectified - 8 | No error - 4 | No error - 8 | No error - 8 | No error - 8 | 64 |
| 79 | 3D | B | 3 | Error not rectified - 6 | No error - 2 | No error - 2 | No error - 2 | No error - 2 | No error - 2 | Error rectified - 8 | No error - 4 | No error - 4 | No error - 4 | No error - 4 | No error - 8 | No error - 8 | No error - 8 | 64 |
| 80 | 4K | A | 2 | No error - 2 | No error - 2 | No error - 2 | No error - 2 | No error - 2 | No error - 2 | No error - 4 | No error - 4 | No error - 4 | No error - 4 | No error - 4 | No error - 8 | No error - 8 | No error - 8 | 56 |
| 81 | 3D | A | 2 | No error - 2 | No error - 2 | Error rectified - 4 | No error - 2 | No error - 2 | No error - 2 | No error - 4 | No error - 4 | No error - 4 | No error - 4 | No error - 4 | No error - 8 | No error - 8 | No error - 8 | 58 |
| 82 | 4K | B | 2 | Error not rectified - 6 | Error not rectified - 6 | No error - 2 | No error - 2 | No error - 2 | No error - 2 | No error - 4 | No error - 4 | No error - 4 | No error - 4 | No error - 4 | No error - 8 | No error - 8 | No error - 8 | 64 |
| 83 | 3D | A | 2 | Error not rectified - 6 | No error - 2 | No error - 2 | Error rectified - 4 | No error - 2 | No error - 2 | No error - 4 | No error - 4 | No error - 4 | No error - 4 | No error - 4 | No error - 8 | No error - 8 | No error - 8 | 62 |
| 84 | 4K | A | 2 | No error - 2 | No error - 2 | No error - 2 | No error - 2 | No error - 2 | No error - 2 | No error - 4 | No error - 4 | No error - 4 | No error - 4 | No error - 4 | No error - 8 | No error - 8 | No error - 8 | 56 |
| 85 | 4K | C | 2 | Error rectified - 4 | Error not rectified - 6 | No error - 2 | No error - 2 | No error - 2 | No error - 2 | No error - 4 | No error - 4 | No error - 4 | No error - 4 | No error - 4 | No error - 8 | No error - 8 | No error - 8 | 62 |
| 86 | 3D | B | 2 | Error rectified - 4 | No error - 2 | No error - 2 | No error - 2 | No error - 2 | No error - 2 | Error rectified - 8 | No error - 4 | No error - 4 | No error - 4 | No error - 4 | No error - 8 | No error - 8 | No error - 8 | 62 |
| 87 | 3D | B | 3 | Error rectified - 4 | No error - 2 | No error - 2 | No error - 2 | No error - 2 | No error - 2 | Error rectified - 8 | No error - 4 | No error - 4 | No error - 4 | No error - 4 | No error - 8 | No error - 8 | No error - 8 | 62 |
| 88 | 3D | A | 3 | No error - 2 | No error - 2 | No error - 2 | No error - 2 | No error - 2 | No error - 2 | No error - 4 | No error - 4 | No error - 4 | No error - 4 | No error - 4 | No error - 8 | No error - 8 | No error - 8 | 56 |
| 89 | 3D | B | 2 | No error - 2 | No error - 2 | No error - 2 | No error - 2 | No error - 2 | No error - 2 | No error - 4 | No error - 4 | No error - 4 | No error - 4 | No error - 4 | No error - 8 | No error - 8 | No error - 8 | 56 |
| 90 | 4K | B | 2 | No error - 2 | No error - 2 | No error - 2 | No error - 2 | No error - 2 | No error - 2 | No error - 4 | No error - 4 | No error - 4 | No error - 4 | No error - 4 | No error - 8 | No error - 8 | No error - 8 | 56 |
| 91 | 4K | A | 2 | Error not rectified - 6 | Error not rectified - 6 | No error - 2 | No error - 2 | No error - 2 | No error - 2 | No error - 4 | No error - 4 | No error - 4 | No error - 4 | No error - 4 | No error - 8 | No error - 8 | No error - 8 | 64 |
| 92 | 4K | A | 3 | Error not rectified - 6 | No error - 2 | No error - 2 | No error - 2 | No error - 2 | No error - 2 | No error - 4 | No error - 4 | No error - 4 | Error rectified - 8 | No error - 4 | No error - 8 | No error - 8 | No error - 8 | 64 |
| 93 | 3D | A | 2 | Error not rectified - 6 | No error - 2 | No error - 2 | No error - 2 | No error - 2 | No error - 2 | No error - 4 | No error - 4 | No error - 4 | No error - 4 | No error - 4 | No error - 8 | No error - 8 | No error - 8 | 60 |
| 94 | 4K | B | 3 | Error not rectified - 6 | No error - 2 | No error - 2 | No error - 2 | No error - 2 | No error - 2 | No error - 4 | No error - 4 | No error - 4 | No error - 4 | No error - 4 | No error - 8 | No error - 8 | No error - 8 | 60 |
| 95 | 4K | A | 3 | No error - 2 | No error - 2 | No error - 2 | No error - 2 | No error - 2 | No error - 2 | No error - 4 | No error - 4 | No error - 4 | No error - 4 | No error - 4 | No error - 8 | No error - 8 | No error - 8 | 56 |
| 96 | 4K | A | 3 | No error - 2 | No error - 2 | No error - 2 | No error - 2 | No error - 2 | No error - 2 | No error - 4 | No error - 4 | No error - 4 | No error - 4 | No error - 4 | No error - 8 | No error - 8 | No error - 8 | 56 |
| 97 | 4K | A | 1 | No error - 2 | No error - 2 | No error - 2 | No error - 2 | No error - 2 | No error - 2 | No error - 4 | No error - 4 | No error - 4 | No error - 4 | No error - 4 | No error - 8 | No error - 8 | No error - 8 | 56 |
| 98 | 3D | C | 2 | No error - 2 | No error - 2 | No error - 2 | No error - 2 | No error - 2 | No error - 2 | No error - 4 | Error rectified - 8 | No error - 4 | No error - 4 | No error - 4 | No error - 8 | No error - 8 | No error - 8 | 60 |
| 99 | 3D | C | 2 | Error rectified - 4 | No error - 2 | No error - 2 | Error rectified - 4 | Error rectified - 4 | No error - 2 | No error - 4 | No error - 4 | No error - 4 | No error - 4 | No error - 4 | No error - 8 | No error - 8 | No error - 8 | 62 |
| 100 | 4K | C | 2 | No error - 2 | No error - 2 | No error - 2 | No error - 2 | No error - 2 | Error rectified - 4 | No error - 4 | No error - 4 | No error - 4 | Error rectified - 8 | No error - 4 | No error - 8 | No error - 8 | No error - 8 | 62 |
| 101 | 3D | C | 2 | No error - 2 | No error - 2 | No error - 2 | Error rectified - 4 | No error - 2 | No error - 2 | No error - 4 | No error - 4 | No error - 4 | Error rectified - 8 | No error - 4 | No error - 8 | No error - 8 | No error - 8 | 62 |
| 102 | 3D | C | 2 | No error - 2 | No error - 2 | No error - 2 | Error rectified - 4 | No error - 2 | No error - 2 | No error - 4 | No error - 4 | No error - 4 | Error rectified - 8 | No error - 4 | No error - 8 | No error - 8 | No error - 8 | 62 |
| 103 | 3D | B | 2 | No error - 2 | No error - 2 | No error - 2 | No error - 2 | No error - 2 | No error - 2 | No error - 4 | No error - 4 | No error - 4 | No error - 4 | No error - 4 | No error - 8 | No error - 8 | No error - 8 | 56 |
| 104 | 4K | A | 3 | No error - 2 | No error - 2 | No error - 2 | No error - 2 | No error - 2 | No error - 2 | No error - 4 | No error - 4 | No error - 4 | No error - 4 | No error - 4 | No error - 8 | No error - 8 | No error - 8 | 56 |
| 105 | 4K | B | 3 | No error - 2 | No error - 2 | No error - 2 | No error - 2 | No error - 2 | No error - 2 | No error - 4 | No error - 4 | No error - 4 | No error - 4 | No error - 4 | No error - 8 | No error - 8 | No error - 8 | 56 |
| 106 | 3D | B | 3 | Error rectified - 4 | No error - 2 | Error rectified - 4 | Error rectified - 4 | No error - 2 | No error - 2 | No error - 4 | No error - 4 | No error - 4 | No error - 4 | No error - 4 | No error - 8 | No error - 8 | No error - 8 | 62 |
| 107 | 4K | A | 2 | No error - 2 | No error - 2 | Error rectified - 4 | No error - 2 | No error - 2 | No error - 2 | No error - 4 | No error - 4 | No error - 4 | No error - 4 | No error - 4 | No error - 8 | No error - 8 | No error - 8 | 58 |
| 108 | 3D | A | 3 | No error - 2 | No error - 2 | No error - 2 | No error - 2 | No error - 2 | No error - 2 | No error - 4 | No error - 4 | No error - 4 | No error - 4 | No error - 4 | No error - 8 | No error - 8 | No error - 8 | 56 |
| 109 | 3D | B | 2 | No error - 2 | No error - 2 | No error - 2 | No error - 2 | No error - 2 | No error - 2 | No error - 4 | No error - 4 | No error - 4 | No error - 4 | No error - 4 | No error - 8 | No error - 8 | No error - 8 | 56 |

*Pt. = Patient, Cons. = Consultant*
